# Synthesis of mesomeric betaine compounds with imidazolium-enolate structure

**DOI:** 10.3762/bjoc.8.42

**Published:** 2012-03-13

**Authors:** Nina Gonsior, Fabian Mohr, Helmut Ritter

**Affiliations:** 1 Institut für Organische Chemie und Makromolekulare Chemie, Heinrich-Heine-Universität Düsseldorf, Universitätsstraße 1, 40225 Düsseldorf, Germany; 2 Fachbereich C - Anorganische Chemie, Bergische Universität Wuppertal, Gaußstr. 20, 42119 Wuppertal, Germany

**Keywords:** cyclodextrin, heterocyclic mesomeric betaine, imidazole, polymer, X-ray single-crystal analysis

## Abstract

The synthesis of a heterocyclic mesomeric betaine by quaternization reaction of 1-butylimidazole and tetrabromo-1,4-benzoquinone is presented. The structure was verified by means of X-ray single-crystal analysis, NMR and IR spectroscopy. Inclusion complexes of the heterocyclic mesomeric betaine with randomly methylated (1.8) β-cyclodextrin were investigated by UV–vis spectroscopy. Furthermore, the reaction conditions were applied to poly(vinylimidazole) and 1,4-bis(1*H*-imidazol-1-yl)butane to obtain functionalized polymer networks and condensate polymers, respectively.

## Introduction

Heterocyclic mesomeric betaines [1] are interesting starting materials for heterocycle and polymer synthesis due to there intriguing chemical properties. They can be broadly classified into four main groups, i.e., conjugated heterocyclic mesomeric betaines (CMB), which are associated with 1,3-dipoles; cross-conjugated heterocyclic mesomeric betaines (CCMB), which are associated with 1,4-dipoles; pseudo-cross-conjugated mesomeric betaines (PCCMB), which can be converted into Arduengo carbens [2–3]; and *N*-ylides, which form a subclass of CMB [1]. In the field of polymer science, CMBs such as pyridiniumolates and isoquinolinium-3-olates, as well as CCMB-systems based on pyrimidiniumolates, are mainly used as photosensitive materials. Different types of mesoionic monomers containing styrenic [4–6] or methacrylic [7–8] moieties were synthesized and polymerized by Ritter et al. Furthermore, photosensitive mesoionic main-chain polymers were also prepared [9–10]. Recently, A. Schmidt et al. described the synthesis of polymeric mesomeric betaines by quaternization of poly(4-vinylpyridine) with different halide-containing quinone derivatives and subsequent hydrolysis [11]. Since the quaternization reaction is very common for imidazole chemistry, e.g., synthesis of ionic liquids, we investigated the quaternization reaction of three different imidazole compounds with tetrabromo-1,4-bezoquinone to obtain novel mesomeric betaine materials with imidazolium-enolate structure.

## Results and Discussion

**Scheme 1:**
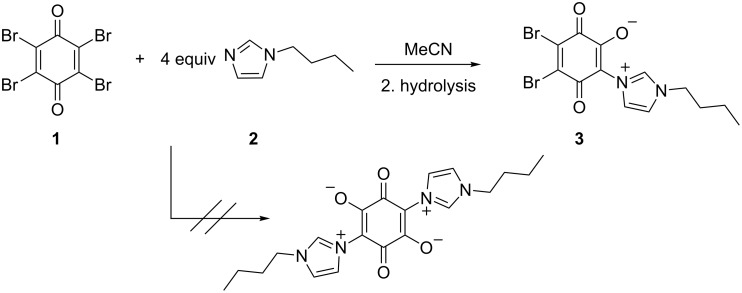
Reaction of p-bromanil (**1**) with 1-butylimidazole (**2**).

The quaternization of 1-butylimidazole (**2**) with tetrabromo-1,4-benzoquinone (*p*-bromanil (**1**)) in acetonitrile and the subsequent quenching of the reaction mixture with water yielded dipole 2,3-dibromo-5-(1-butyl-1*H*-imidazol-3-ium-3-yl)-1,4-benzoquinone-6-olate (**3**) instead of the quadrupole compound (Scheme 1). Model compound **3** was dark red, solid, and soluble in chloroform, dichloromethane, and DMSO, but insoluble in water, acetone and toluene. The formation of **3** was confirmed by means of NMR and IR spectroscopy, as well as X-ray single-crystal analysis.

**Figure 1:**
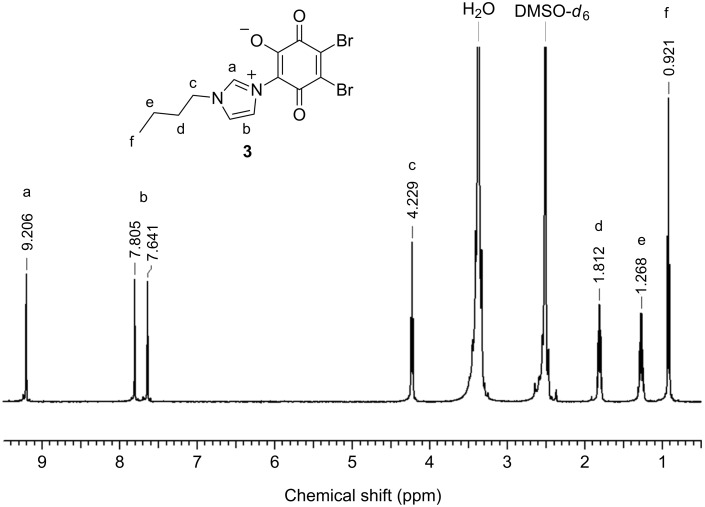
^1^H NMR spectrum of mesomeric betaine **3**.

The ^1^H NMR spectrum of **3** is depicted in Figure 1. The signals for all structural features were found, e.g., at 9.21 ppm, 7.81 ppm and 7.64 ppm, for the imidazolium protons, and in the range of 4.23–0.92 ppm for the butyl moiety.

**Figure 2:**
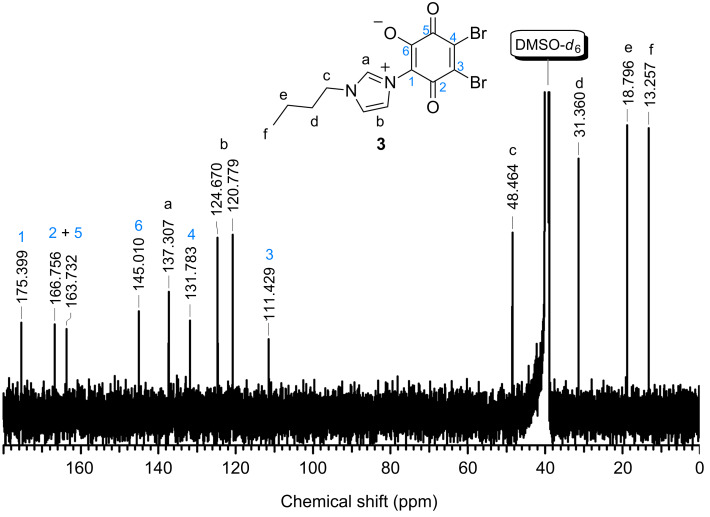
^13^C NMR spectrum of dipole **3**.

Furthermore, the ^13^C NMR spectrum confirms the exclusive formation of dipole **3** and the absence of the quadrupole (Figure 2). For the dipole formation, six individual carbon signals for the benzoquinone moiety are recorded, whereas only three signals should be obtained in case of the quadrupole, due to the molecule symmetry. The characteristic peaks of imidazolium appeared at 137.3 ppm, 124.7 ppm, and 120.8 ppm. The peaks of the benzoquinone carbons were recorded in the range of 175.4 to 111.4 ppm, whereas the signals for the butyl group appeared in the range from 48.5 to 13.3 ppm.

**Figure 3:**
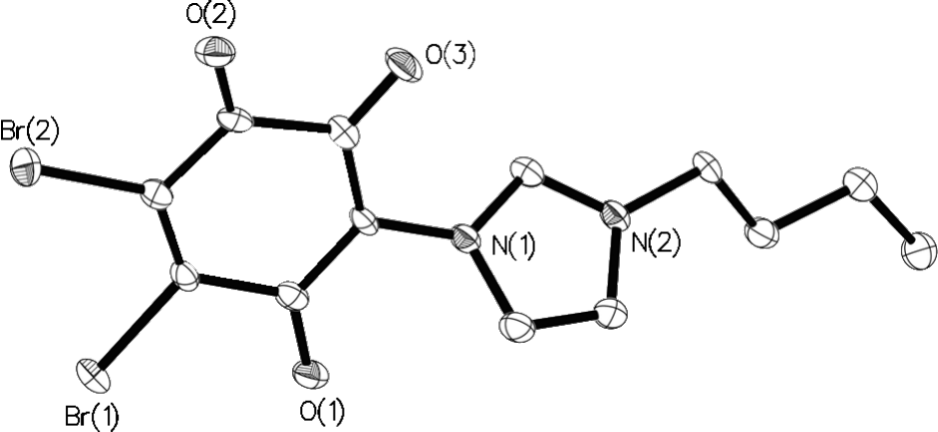
Solid-state molecular structure of compound **3**. Selected bond distances [Å]: O(1)–C 1.228(4), O(2)–C 1.204(4), O(3)–C 1.241(4).

Furthermore, the specific O–C–C–C–O vibration band was found at 1540 cm^−1^ in the IR spectra. By means of X-ray single-crystal analysis, the structure of **3** was clearly identified (Figure 3). Single crystals were obtained by the evaporation method, in which methylene chloride was used as the solvent.

**Figure 4:**
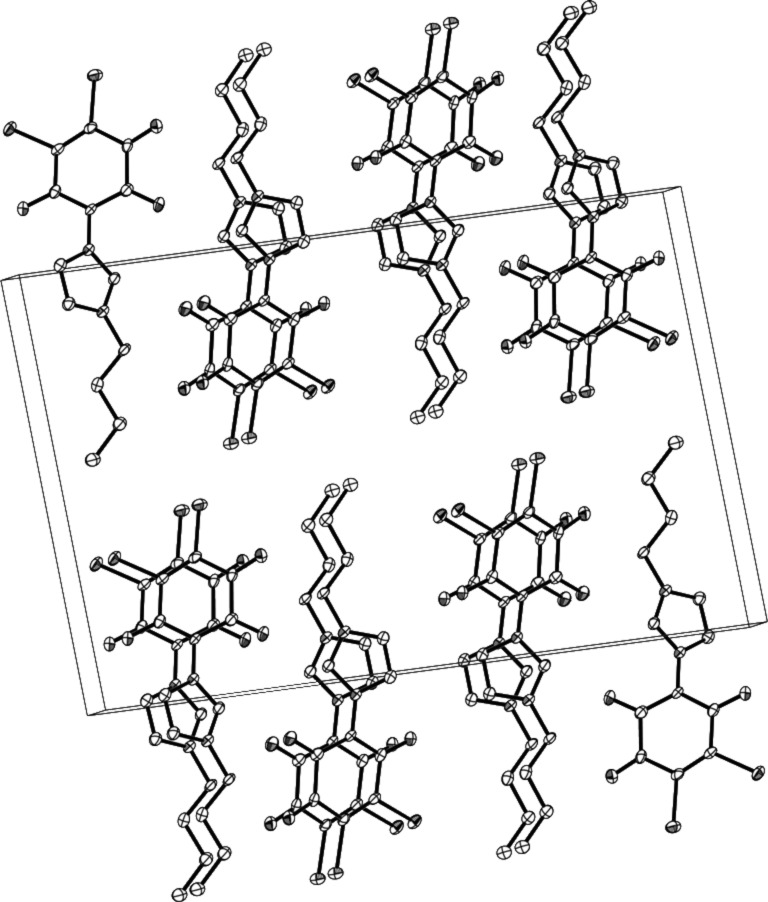
Formation of molecular layers in the crystal packing.

The compound crystallized in the space group *P*21/*c* (No. 14). The bond distances of O(3)–C and O(1)–C were 1.241(4) Å and 1.228(4) Å, respectively. These correspond to the values of the C=O double bonds. In the crystal packing, molecule layers were formed due to very weak π–π stacking of the aromatic rings (Figure 4).

**Figure 5:**
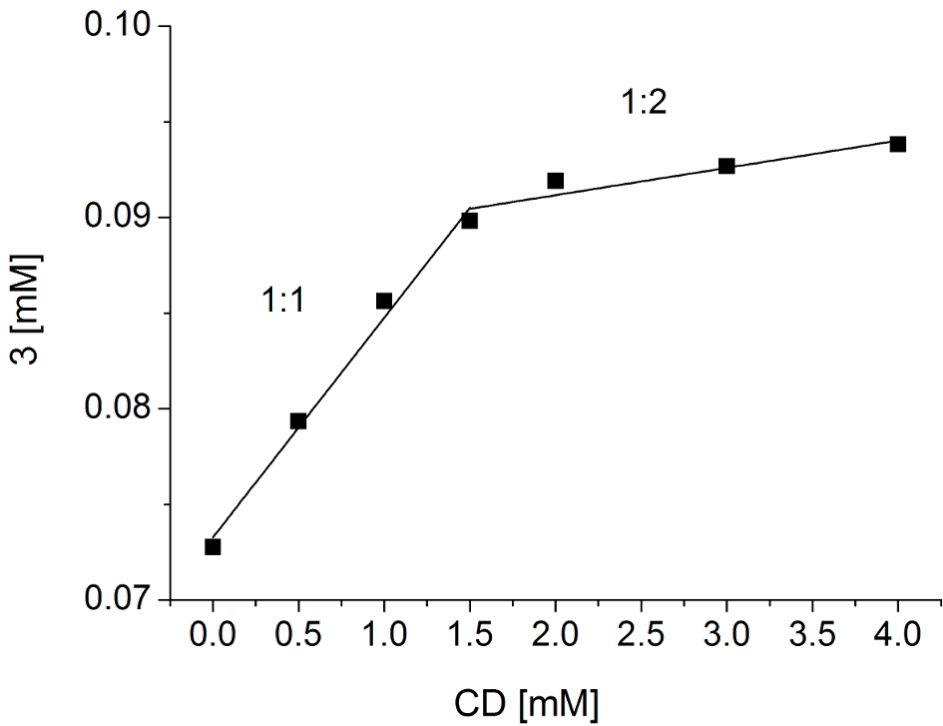
Higuchi–Connors phase diagram of **3**/m-β-CD complex.

Regarding our interest in supramolecular chemistry and the enhancement of water solubility, the ability of **3** to form inclusion complexes with randomly methylated (1.8) β-cyclodextrin (m-β-CD) was investigated by means of UV–vis spectroscopy. Therefore, the type of inclusion complex and the complex formation constant (*K*) were investigated, based on the phase-solubility technique. The Higuchi–Connors phase diagram [12] indicates an initial 1:1 complex, while at higher concentrations a 1:2 complex is formed (Figure 5, for concentrations used for UV–vis measurements, see Supporting Information File 1).

**Scheme 2:**
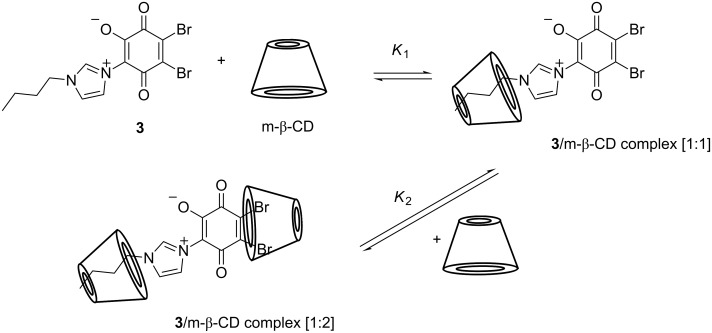
Mechanism of molecular association of the complex.

The relatively low values of the formation constant calculated for both a 1:1 complex (*K*_1_ = 1.6 × 10^2^ M^−1^) and a 1:2 complex (*K*_2_ = 0.2 × 10^2^ M^−1^) indicates only weak host/guest interactions and therefore only a slight increase of solubility in water was achieved. However, the value of *K*_1_ is in good agreement with those already reported for 1-butylimidazole (1.54 × 10^2^ M^−1^) [13]. Thus, it can be assumed that the inclusion formation follows the suggested mechanism depicted in Scheme 2.

**Figure 6:**
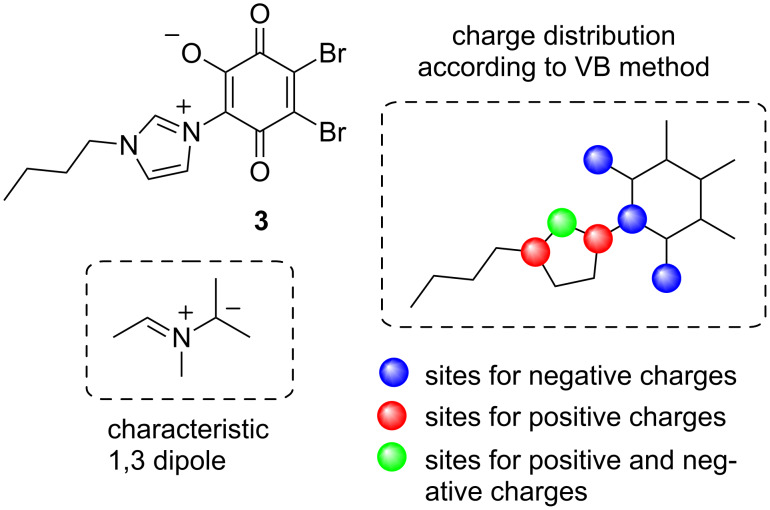
Classification of betaine **3**.

Based on the comprehensive classification system for heterocyclic mesomeric betaines proposed by Ollis, Stanforth, and Ramsden in 1985 [1], mesoion **3** belongs to the class of conjugated heterocyclic mesomeric betaines (CMB). According to the valence-bond (VB) approach for the classification, it was found that the positive and negative charges are in mutual conjugation and both are associated with the common conjugated π-electron system of the molecule [1]. An alternative method to classify this type of conjugation is the recognition of characteristic 1,3-dipole increments from canonical formulae. Figure 6 summarizes both types of classification for the CMB compound **3**.

**Scheme 3:**
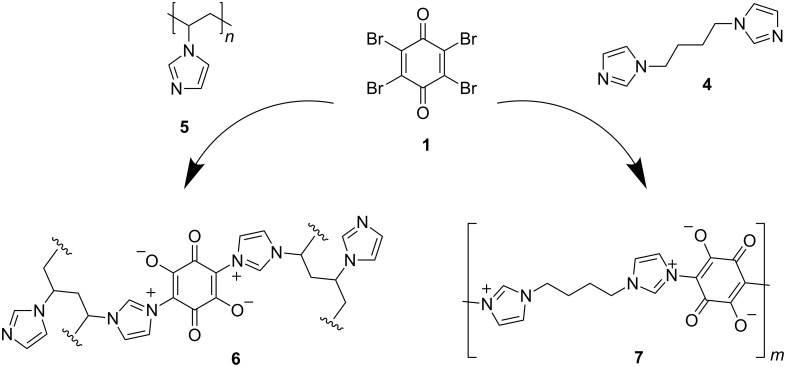
Synthesis of polymer **6** and oligomer **7** based on imidazolium-enolate structures.

According to our interests, mesoion **3** was synthesized as a model compound for further application in the field of polymer chemistry (Scheme 3). Therefore, *p*-bromanil (**1**) was reacted with different proportions of poly(vinylimidazole) (**5**) (a = 1:4, b = 1:20, c = 1:40) to obtain polymer networks, which can be represented by the idealized structure **6**. Each polymer network **6a**–**c** was insoluble in organic solvents. The nitrogen contents determined by elemental analysis showed that with an increasing amount of **5** the nitrogen content also increased [**6a** N: 18.49; **6b** N: 21.99; **6c** N: 23.83]. Furthermore, the intensity of the specific O–C–C–C–O vibration band at 1539 cm^−1^ decreased in the order of **6a** > **6b** > **6c**, which corresponds to a looser network structure for **6c** compared to **6a**. However, it is not possible to distinguish between inter- and intramolecular cross-linkings. To obtain condensate polymer **7**, **1** was reacted with 1,4-bis(1*H*-imidazol-1-yl)butane (**4**). However, the reaction with both monomer balance (**a**) and imbalance (**b**) only led to oligomeric compounds (**7a**,**b**), which were soluble in water and DMSO but insoluble in, e.g., CHCl_3_, acetone or toluene. MALDI-TOF mass spectroscopy revealed a maximum repeat unit of *m* = 3 with 1,4-bis(1*H*-imidazol-1-yl)butane end-groups on both sides of the chain. The specific O–C–C–C–O vibration band was found at 1541 cm^−1^ in the IR spectra.

**Figure 7:**
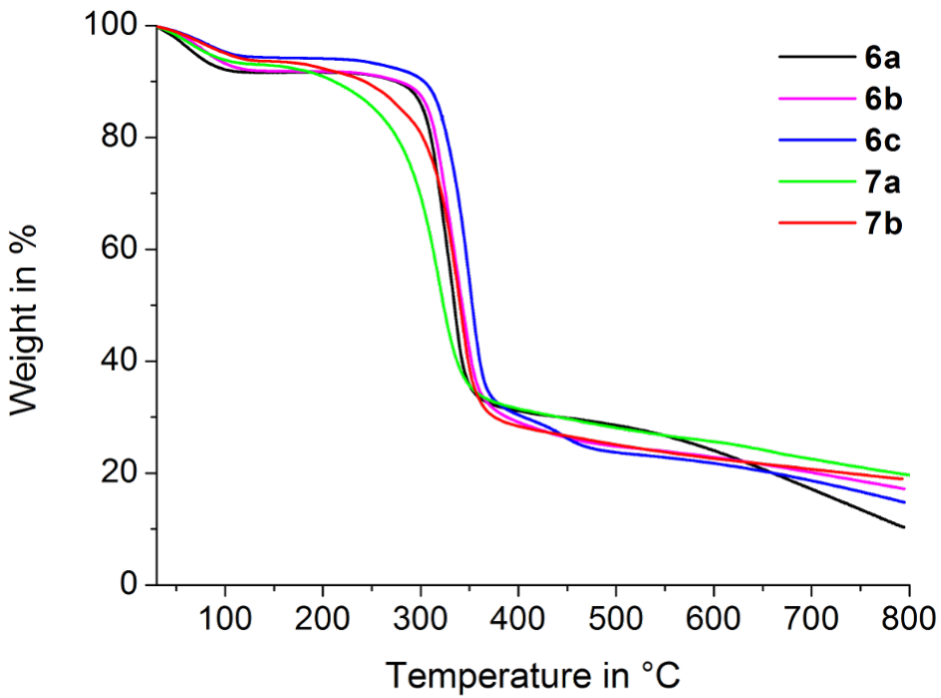
Thermogravimetric analyses of polymer networks **6a–c** and oligomers **7a**,**b**.

Thermogravimetric analyses are depicted in Figure 7. The weight losses of up to 10% at temperatures lower than 120 °C are due to the extrusion of water. At higher temperatures unidentified decomposition was obtained, since the extrusion of one component results in the destruction of the polymer backbone. However, further conclusions can be drawn from the thermal stability. Polymers **6a**–**c** are stable up to 300 °C, while oligomers **7a** and **7b** decompose at temperatures between 200 and 220 °C. Furthermore, considering the temperatures corresponding to 50% weight loss, the thermal stability decreases in the order of **6c** (352 °C) > **6b** (343 °C) > **7b** (340 °C) > **6a** (334 °C) > **7a** (320 °C).

## Conclusion

The synthesis of novel heterocyclic mesomeric betaine **3** by quaternization reaction of 1-butyl-1*H*-imidazole and tetrabromo-1,4-benzoquinone was presented. The structure was verified by means of X-ray single-crystal analysis, as well as NMR and IR spectroscopy. Furthermore, mesoion **3** was classified by the comprehensive classification system for heterocyclic mesomeric betaines, as a conjugated mesomeric betaine. Inclusion complexes of **3** with m-β-CD were investigated by UV–vis spectroscopy. The Higuchi–Connors phase diagram indicated the formation of weak 1:1 and 1:2 complexes. In addition, the reaction conditions were applied to poly(vinylimidazole) and 1,4-bis(1*H*-imidazol-1-yl)butane to obtain functionalized polymer networks and oligomers, which were stable at temperatures up to 300 °C and 200 °C, respectively.

## Experimental

**Materials.** Randomly methylated (1.8) β-cyclodextrin (m-β-CD) was obtained from Wacker-Chemie GmbH (Burghausen, Germany). Prior to use, m-β-CD was dried in a CEM Sam 255 microwave drying system and stored in a desiccator under vacuum over sicapent. 1-Butylimidazole (98%), 1,4-dichlorobutane (97%), and azobisisobutyronitrile (98%) were obtained from Aldrich Chemicals (Germany). 1-Vinylimidazole (99%) and tetrabromo-1,4-benzoquinone (*p*-bromanil) were purchased from Alfa Aesar and 1-*H*-imidazole was obtained from AppliChem (Germany). Dimethylsulfoxide-*d*_6_ (DMSO-*d*_6_) 99.8 atom % D was purchased from Eurisotop^®^ (France). If not stated otherwise, all commercially available reagents and solvents were used without further purification.

**Measurements.** The structures of the synthesized compounds were evaluated by ^1^H and ^13^C NMR spectroscopy by using a Bruker Avance DRX 500 spectrometer at 500.13 MHz for proton and 125.77 MHz for carbon. With DMSO-*d*_6_ as solvent, chemical shifts were referenced to the solvent values at δ_H_ = 2.51 ppm and δ_C_ = 39.52 ppm. C, H and N elemental analysis was determined by using a Perkin Elmer 2400 CHN analyzer. Infrared spectra were recorded on a Nicolet 6700 FT-IR spectrometer equipped with a diamond single-bounce ATR accessory at room temperature. Thermogravimetric analyses (TGA) were carried out with a TA 600 Perkin Elmer (TGA combined with a DTA) in a temperature range between 30 and 800 °C under an argon atmosphere. The heating rate was 10 °C min^−1^. All measurements were baseline corrected and analyzed by Pyris software. Determination of the type of inclusion complex and the complexation constant was achieved by UV–vis spectroscopy. The UV–vis spectra were recorded on a Nicolet UV540 spectrometer in the range from 190 to 400 nm in a glass cuvette with a layer thickness of 1 cm. Furthermore, the change of absorbance at a wavelength of λ = 306 nm was determined according to the cyclodextrin concentration. Matrix-assisted laser desorption/ionization-time-of-flight mass spectrometry (MALDI-TOF-MS) was performed on a Bruker Ultraflex TOF mass spectrometer. Ions formed with a pulsed nitrogen laser (25 Hz, 337 nm) were accelerated to 25 kV, with the molecular masses being recorded in the linear mode. 2,5-Dihydroxybenzoic acid (DBH) in acetonitrile/water (25 mg mL^−1^) was used as a matrix. The samples (1 mg mL^−1^ in water) were mixed with the matrix solution at a volumetric ratio of 1:2. Molecular weights and molecular weight distributions were measured by size-exclusion chromatography (SEC) with a Viscotek GPCmax VE2001 system that contained a column set up with one Viscotek TSK guard column HHR-H 6.0 mm (ID)_4 cm (L) and two Viscotek TSK GMHHR-M 7.8 mm (ID)_30 cm (L) columns at 60 °C. *N*,*N*-Dimethylformamide (DMF, 0.1 M LiCl) was used as eluent at a flow rate of 1 mL min^−1^. A Viscotek VE 3500 RI detector and a Viscotek Viscometer model 250 were used. The system was calibrated with polystyrene standards with a molecular range from 580 Da to 1186 kDa.

### Synthesis of 2,3-dibromo-5-(1-butyl-1*H*-imidazol-3-ium-3-yl)-1,4-benzoquinone-6-olate (**3**)

Tetrabromo-1,4-benzoquinone (*p*-bromanil, **1**) (0.845 g, 2 mmol) was dissolved in 45 mL of acetonitrile at 60 °C and 1-butyl-1*H*-imidazole (**2**) (1 g, 8 mmol) was added. The mixture was heated under reflux for 3 h and was afterwards hydrolyzed with 40 mL of water. After filtration, acetonitrile was removed by evaporation and the resulting solution was cooled at 0 °C until a precipitate was formed. The crude product was collected by filtration and was washed three times with 50 mL of water. After recrystallization from acetic acid, **3** was obtained as a dark red solid. Yield: 247 mg (0.61 mmol, 31%); ^1^H NMR (500 MHz, DMSO-*d*_6_) δ 9.11 (s, 1H, N(1)C*H*N(3)), 7.816 (s, 1H, N(1)CHC*H*), 7.64 (s, 1H, N(1)C*H*CH), 4.23 (t, ^3^*J*_H,H_ = 7.09 Hz, 2H, N(1)C*H_2_*), 1.81 (m, 2H, N(1)CH_2_C*H_2_*), 1.27 (m, 2H, C*H_2_*CH_3_), 0.92 (t, ^3^*J*_H,H_ = 7.41 Hz, 3H, CH_2_C*H_3_*) ppm; ^13^C NMR (125 MHz, DMSO-*d*_6_) δ 175.39 (N(3)–*C*(CO)CO), 166.76, 163.73 (*C*=O), 145.01 (*C*O), 137.31 (N(1)*C*HN(3)), 131.78 (*C*–Br), 124.67 (N(1)CH*C*H), 120.78 (N(1)*C*HCH), 111.43 (*C*–Br), 48.46 (N(1)*C*H_2_), 31.36 (N(1)CH_2_*C*H_2_), 18.79 (*C*H_2_–CH_3_), 13.26 (CH_2_–*C*H_3_) ppm; IR (cm^−1^): 3185, 3128, 3066 (CH, imidazole), 2960, 2915, 2877 (CH_3_, CH_2_), 1697 (C=O), 1569, 1552 (C=C_arom_), 1540 (O–C–C–C–O), 1469, 1338 (δ CH_3_, CH_2_), 1149 (HCC, HCN bending, imidazole), 850, 736, 582; Anal. calcd for C_13_H_12_Br_2_N_2_O_3_: C, 38.64; H, 2.99; N, 6.93; found: C, 38.65; H, 2.86; N, 6.59.

### X-ray crystal-structure analysis of **3**

Single crystals of **3** were obtained by very slow evaporation of methylene chloride. They were mounted in inert oil and transferred to the cold gas stream of the diffractometer. Diffraction data were collected at 150 K by using an Oxford Diffraction Gemini E Ultra diffractometer [Cu Kα irradiation (λ = 1.5418 Å)], with an EOS CCD area detector and a four-circle kappa goniometer. Data integration, scaling and empirical absorption correction was carried out with the CrysAlis Pro program package [14]. The structure was solved by using Direct Methods and refined by Full-Matrix-Least-Squares against F^2^. The non-hydrogen atoms were refined anisotropically and hydrogen atoms were placed at idealized positions and refined by using a model riding mode. All calculations were carried out with the program Olex2 [15]. Brief summary of crystal data: C_13_H_12_Br_2_N_2_O_3_, *M* = 404.07, Monoclinic, *a* = 15.0119(6) Å, *b* = 3.99067(18) Å, *c* = 22.8802(9) Å, β = 93.428(4)°, *V* = 1368.24(10) Å^3^, space group *P*21/*c* (No. 14), *Z* = 4, μ(Cu Kα) = 7.611, 4645 reflections measured, 2161 unique (*R*_int_ = 0.0437), which were used in all calculations. The final *wR*(*F*_2_) was 0.0921 (all data). All important crystallographic data, refinement details, bond lengths, and bond angles for **3** are summarized in Supporting Information File 2.

### Synthesis of 1,4-bis(1*H*-imidazol-1-yl)butane (**4**)

Compound 4 was synthesized according to the literature [16]. A mixture of 1*H*-imidazole (6.8 g, 0.1 mol) and NaOH (4.0 g, 0.1 mol) was stirred in DMSO (20 mL) at 60 °C for 1 h. 1,4-Dichlorobutane (6.4 g, 0.05 mol) was added and the mixture was stirred for a further 2 h at 60 °C. Afterwards, the reaction mixture was poured into 200 mL of water and a white solid slowly precipitated. The product was collected by filtration, washed three times with water (20 mL) and freeze dried in order to remove the remaining water. Yield**:** 6.18 g (0.032 mol, 65%); ^1^H NMR (500 MHz, DMSO-*d*_6_) δ 7.61 (s, 1H, N(1)C*H*N(3)), 7.14 (s, 1H, N(1)–CHC*H*), 6.88 (s, 1H, N(1)–C*H*CH), 3.96 (m, 2H, CH_2_), 1.62 (m, 2H, CH_2_); ^13^C NMR (125 MHz, DMSO-*d*_6_) δ 137.25 (N(1)*C*HN(3)), 128.43 (N(1)CH*C*H), 119.27 (N(1)*C*HCH), 45.29 (N(1)*C*H_2_), 27.71 (N(1)CH_2_*C*H_2_); IR (cm^−1^): 3390 (OH, water), 3094 (CH, imidazole), 2939, 2860 (CH_2_), 1641 (C=C, C=N), 1508, 1463 (C=C_arom_), 1452, 1393 (δ CH_2_), 1229, 1084, 774, 665; Anal. calcd for C_10_H_14_N_4_·0.5 H_2_O crystal water: C, 60.28; H, 7.59; N, 28.12; found: C, 59.95; H, 7.25; N, 27.99.

### Syntheses of the polymers

#### Poly(vinylimidazole) (**5**)

Vinylimidazole (10 g, 0.106 mol) was dissolved in THF (50 mL) under a nitrogen atmosphere and AIBN (0.44 g, 2.7 mmol) was added as a radical initiator. The reaction mixture was stirred for 16 h at 65 °C. The polymer precipitated during the reaction and the obtained white solid was collected by filtration, washed three times with THF (200 mL) and dried under vacuum. ^1^H NMR (500 MHz, DMSO-*d*_6_) δ 7.46–7.01 (s, 1H, N(1)C(*H*)N(3)), 6.98–6.67 (br, 2H, N(1)–CHCH–N(3)), 3.28–2.79 (s (br), 1H, backbone N(1)CH), 2.28–1.61 (s (br), 2H, backbone-CH_2_); GPC (DMF, PS-Standard) *M*_n_ = 150 000 g mol^−1^.

#### Polymers **6a–c**

*p*-Bromanil (**1**) (250 mg, 0.59 mmol) and poly(vinylimidazole) (**5**) (0.22 g (**a**), 1.11 g (**b**) or 2.22 g (**c**)) were suspended in acetonitrile (45 mL) and heated under reflux for 3 h. The reaction mixture was subsequently diluted with water (40 mL) for the hydrolysis reaction. The crude product was collected by filtration, washed several times with water and was heated under reflux with acetone for 1 h. After filtration and washing with acetone, polymers **6a**–**c** were obtained as an orange or brownish solid. IR (cm^−1^): 3388 (OH, water), 3102 (CH, imidazole), 2939 (CH_2_), 1632, 1538 (O–C–C–C–O), 1494, 1414 (δ CH_2_), 1227, 1084, 814, 742, 662. **6a**: Anal. found: C, 45.02; H, 5.29; N, 18.49. **6b**: Anal. found: C, 51.26; H, 5.35; N, 21.99. **6c**: Anal. found: C, 54.32; H, 5.93; N, 23.83.

#### Synthesis of Oligomer **7** with both, monomer balance (**7a**) and monomer imbalance (**7b**)

*p*-Bromanil (**1**) (557 mg, 1.31 mmol) and 1,4-bis(1*H*-imidazol-1-yl)butane (**4**) (1 equiv (**a**) and 2 equiv (**b**), respectively) were dissolved in 45 mL of acetonitrile at 60 °C and heated to reflux for 20 h. The reaction mixture was subsequently diluted with water (40 mL) to hydrolyze the product. The solvents were evaporated and the obtained solid was redissolved in methanol. After precipitation in diethyl ether **7a**, **7b** were collected by filtration and dried in vacuum to give red solids. ^1^H NMR (500 MHz, DMSO-*d*_6_) δ 9.27 (s, 1H, N(1)C*H*N(3)), 7.84 (s, 1H, N(1)CHC*H*), 7.33 (s, 1H, N(1)C*H*CH), 4.28 (m, 2H, N(1)C*H_2_*), 1.81 (m, 2H, N(1)CH_2_C*H_2_*) ppm; IR (cm^−1^): 3380 (OH, water), 3091 (CH, imidazole), 2933, 2840 (CH_2_), 1691 (C=O), 1567 (C=C_arom_), 1541 (O–C–C–C–O), 1444, 1384 (δ CH_2_), 1147 (HCC, HCN bending, imidazole), 842, 748.

## Supporting Information

File 1 UV–vis and crystallographic data.

File 2 X-ray structure.
