# Supplementary material for: Synthesis of mesomeric betaine compounds with imidazolium-enolate structure
Source: Beilstein J Org Chem. 2012 Mar 13;8:390–7. doi: 10.3762/bjoc.8.42 (PMC3326615; doi:10.3762/bjoc.8.42)
Supplement: File 1 — UV–vis and crystallographic data. [file Beilstein_J_Org_Chem-08-390-s001.pdf]

Supporting Information

for

Synthesis of mesomeric betaine compounds with imidazolium-enolate structure

Nina Gonsior[1], Fabian Mohr[2] and Helmut Ritter[*1]

Address: [1]Institut für Organische Chemie und Makromolekulare Chemie, Heinrich-Heine-Universität Düsseldorf, Universitätsstraße 1, 40225 Düsseldorf, Germany and [2]Fachbereich C - Anorganische Chemie, Bergische Universität Wuppertal, Gaußstr. 20, 42119 Wuppertal, Germany

Email: Helmut Ritter* - h.ritter@uni-duesseldorf.de

*Corresponding author

UV–vis and crystallographic data

Table of contents

Table 1 and Figure 1 S2

Table 2 S3

Table 3 S4

Table 4 S5

Table 1: Concentration of mesomeric betaine compound **3** and randomly methylated (1.8) β-cyclodextrin (m-β-CD) used for UV–vis measurements.

concentration [mmol·L^{-1}]		molar ratio
**3**	**m-β-CD**	
2	0	1:0
2	0.5	1:0.25
2	1	1:0.5
2	1.5	1:0.75
2	2	1:1
2	3	1:1.5
2	4	1:2

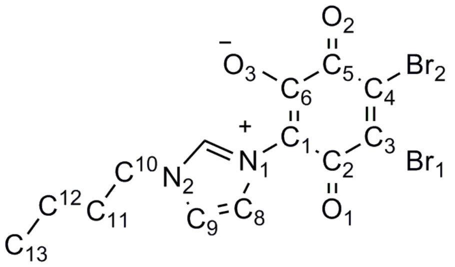

Figure 1: Numbering for the assignment of the bond lengths and angles.

Table 2: Bond lengths for **3** (the numbering corresponds to that in Figure 1).

atom	atom	length [Å]	atom	atom	length [Å]
Br1	C3	1.874(3)	C9	C8	1.345(5)
Br2	C4	1.868(3)	C4	C3	1.337(5)
O3	C6	1.241(4)	C4	C5	1.503(5)
O2	C5	1.204(4)	C2	C1	1.424(5)
N2	C9	1.379(4)	C2	C3	1.514(5)
N2	C7	1.320(4)	C12	C11	1.529(5)
N2	C10	1.493(4)	C12	C13	1.521(5)
O1	C2	1.228(4)	C1	C6	1.398(5)
N1	C7	1.349(4)	C6	C5	1.544(5)
N1	C8	1.379(5)	C10	C11	1.505(5)
N1	C1	1.431(4)			

Table 3: Bond angles for **3** (the numbering corresponds to that in Figure 1).

atom	atom	atom	angle [°]	atom	atom	atom	angle [°]
O3	C6	C1	126.3(3)	C7	N2	C9	109.1(3)
O3	C6	C5	116.8(3)	C7	N2	C10	126.1(3)
O2	C5	C4	121.6(3)	C7	N1	C8	107.9(3)
O2	C5	C6	119.9(3)	C7	N1	C1	126.4(3)
N2	C7	N1	108.4(3)	C8	N1	C1	125.7(3)
N2	C10	C11	112.6(3)	C8	C9	N2	107.0(3)
O1	C2	C1	124.7(3)	C1	C2	C3	116.5(3)
O1	C2	C3	118.8(3)	C1	C6	C5	116.8(3)
C9	N2	C10	124.6(3)	C6	C1	N1	118.4(3)
C9	C8	N1	107.5(3)	C6	C1	C2	124.6(3)
C4	C3	Br1	122.2(3)	C10	C11	C12	111.2(3)
C4	C3	C2	123.8(3)	C3	C4	Br2	124.9(3)
C4	C5	C6	118.5(3)	C3	C4	C5	119.2(3)
C2	C1	N1	117.0(3)	C5	C4	Br2	115.9(2)
C2	C3	Br1	114.0(2)	C13	C12	C11	112.5(3)

Table 4: Crystal data and structure refinement for **3**.

	**3**
Empirical formula	$C_{13}H_{12}Br_2N_2O_3$
Colour	dark red
M [g mol^{-1}]	404.07
Crystal system	Monoclinic
Space group	*P*21/*c*
a, *b*, *c* [Å]	15.0119 (6), 3.99067 (18), 22.8802 (9)
α, β/ γ [°]	90.00, 93.428 (4), 90.00
Volume [Å^3]	1368.24 (10)
Z	4
ρ_{calc} [mg mm^{-3}]	1.962
μ [mm^{-1}]	7.611
F (0 0 0)	792
Crystal size [mm^3]	0.1 × 0.02 × 0.02
θ range for data collection [°]	3.87 to 62.43
Reflections collected	4645
Independent reflections	2161
Parameters	182
Goodness-of-fit	1.038
R_1 [I>2σ (I)]	0.0317
wR_2 [all data]	0.0921
Largest diff. peak/hole [e Å^{-3}]	0.709/−0.878
